# Neuroinflammation in schizophrenia: the role of nuclear factor kappa B

**DOI:** 10.1038/s41398-021-01607-0

**Published:** 2021-10-14

**Authors:** Caitlin E. Murphy, Adam K. Walker, Cynthia Shannon Weickert

**Affiliations:** 1 Neuroscience Research Australia, Randwick, NSW 2031 Australia; 2 School of Psychiatry, University of New South Wales, Randwick, NSW 2031 Australia; 3 Drug Discovery Biology Theme, Monash Institute of Pharmaceutical Sciences, Monash University, Parkville, VIC 3052 Australia; 4 Department of Neuroscience and Physiology, Upstate Medical University, Syracuse, NY 13210 USA

**Keywords:** Molecular neuroscience, Neuroscience

## Abstract

Neuroinflammation, particularly in the dorsolateral prefrontal cortex, is well-established in a subset of people with schizophrenia, with significant increases in inflammatory markers including several cytokines. Yet the cause(s) of cortical inflammation in schizophrenia remains unknown. Clues as to potential microenvironmental triggers and/or intracellular deficits in immunoregulation may be gleaned from looking further upstream of effector immune molecules to transcription factors that control inflammatory gene expression. Here, we focus on the ‘master immune regulator’ nuclear factor kappa B (NF-κB) and review evidence in support of NF-κB dysregulation causing or contributing to neuroinflammation in patients. We discuss the utility of ‘immune biotyping’ as a tool to analyse immune-related transcripts and proteins in patient tissue, and the insights into cortical NF-κB in schizophrenia revealed by immune biotyping compared to studies treating patients as a single, homogenous group. Though the ubiquitous nature of NF-κB presents several hurdles for drug development, targeting this key immunoregulator with novel or repurposed therapeutics in schizophrenia is a relatively underexplored area that could aid in reducing symptoms of patients with active neuroinflammation.

## Introduction

Schizophrenia is a severe psychiatric illness that disrupts the normal functioning of the mind and affects roughly 1% of the population worldwide [1]. People with schizophrenia typically suffer from positive symptoms including delusions, hallucinations and disorganised speech, and negative symptoms such as diminished emotional expression and/or lack of motivation [2, 3]. In addition, there is a high prevalence of often profound neurocognitive deficits among patients, most commonly in working memory, attention, problem solving and processing speed [4]. This combination of psychiatric and cognitive symptoms contributes to reduced scholastic and vocational achievements and worse quality of life [5, 6]. As such, the social and economic costs of schizophrenia are substantial and are disproportionate to the disease prevalence, due to the chronic, severe and often treatment-resistant nature of schizophrenia [2, 7, 8]. Better understanding of the aetiology of schizophrenia is needed in order to develop novel treatments that improve clinical and functional outcomes in patients.

One of the most significant insights into the pathophysiology of schizophrenia over the last 10 years has been the identification of inflammation in patients; however, this has not yet led to effective new treatments. The limited success of clinical trials with anti-inflammatories [9] may reflect previous failures to target the correct inflammatory mechanism(s) with available medications. One approach to identifying the correct target(s) at which to aim novel therapies is to start with the known increase in cytokine mRNA and to then examine factors upstream to determine how these are changed. For example, to ask which, how and to what extent molecular switches known to be responsible for turning on these cytokines are changed. This approach may be more informative given that cytokine synthesis is highly regulated at the transcriptional level, and many specific transcription factors controlling cytokine expression have been identified. Further, control over the activity of these transcription factors can be traced back to receptors capable of responding to the external (extracellular) environment. Altered gene expression of one such ‘master’ immune transcription factor, nuclear factor kappa B (NF-κB), co-occurs with increased cytokine mRNA levels in the brains of people with schizophrenia in at least three studies [10–12].

Another reason that anti-inflammatory treatment may not show a high degree of effectiveness or reproducibility is that not everyone with schizophrenia is expected to be inflamed at commencement of anti-inflammatory treatment. Indeed, it is increasingly apparent that some patients are more likely to be in a state of heightened inflammation and to respond to anti-inflammatory treatment than others. Those with more severe symptoms at baseline are more responsive to adjunctive aspirin [9], and one study found that aspirin response in people with schizophrenia differs based on pro- and anti-inflammatory cytokine ratios at baseline [13]. Results from an earlier clinical trial showed that patients who responded to the cyclo-oxygenase 2 inhibitor celecoxib had lower baseline levels of the anti-inflammatory protein sTNFR1 in blood than non-responders [14]. Since few studies into anti-inflammatory adjuvants in schizophrenia have stratified individuals based on their immune status, it may be the case that only a subset of ‘inflamed’ patients respond favourably to anti-inflammatory drugs, and that this accounts for the small overall effect sizes when people with schizophrenia are considered as a single, homogenous patient group [9, 15].

Before considering these two points, we will review the evidence of inflammation as a potential causal or contributing factor in schizophrenia from three approaches: (1) associations between schizophrenia and immune genes, (2) increased inflammatory markers in postmortem brain tissue from people with schizophrenia and (3) abnormal levels of immune molecules in the blood of living patients. Immune activation assessed in living patients has been linked to more severe positive, negative and cognitive symptoms in patients [16–19], indicating a significant role of inflammation in schizophrenia symptomatology. Heightened or chronic neuroinflammation is not a normal or healthy occurrence, and while the pathogenesis of schizophrenia likely involves several interacting contributors [such as psychosocial stress and/or exposure to recreational drugs [20–23], alleviation of inflammation in people with schizophrenia could bring about therapeutic benefit regardless of its cause.

## Inflammation is associated with schizophrenia

### Genetic evidence of inflammation in schizophrenia

One of the most consistent signals from genome-wide association studies of schizophrenia is significant association between the disease and genetic variation in the major histocompatibility region of chromosome 6 [24–28] which encodes molecules involved in immunity and inflammation [29]. Most notably, a strong association between schizophrenia and the complement system gene C4 was found in a genome-wide association study of more than 28,000 schizophrenia cases and 35,000 control cases [30]. Genes in the major histocompatibility region often contain NF-κB-binding sequences in their promoter regions, including several that encode potent pro-inflammatory cytokines such as interleukin (IL)-1, IL-6, IL-8 and tumour necrosis factor (TNF) [31–37]. Genetic associations to schizophrenia mark broad chromosomal regions which may contain many DNA changes (SNPs) that combine to increase risk, and DNA changes linked to schizophrenia are known occur in promoter regions that regulate gene expression. Thus, this represents a transcriptional enhancement mechanism by which NF-κB, activated by environmental cues, could potentially interact with genomic regions that then increase risk for developing schizophrenia. In support of this, some studies have found significant links between variation in these regions, most notably in the gene encoding interleukin (IL)-1, and schizophrenia susceptibility [38–44]. However, other studies have failed to find an association between IL-1 polymorphisms and schizophrenia risk [45–49]. Lack of replication of SNPs associated with schizophrenia are common, and there are likely multiple genes that need to interact with particular environmental cues to then trigger inflammation, such that no single gene will sufficiently explain risk in all cohorts. This perspective highlights the importance of studying brain gene expression of immunoregulatory pathways as a way of detecting which microenvironmental drivers of inflammation may precipitate schizophrenia genetic risk.

### Postmortem evidence of inflammation in schizophrenia

The study of human postmortem brain tissue allows for the direct measurement of ‘immune’ molecules and markers in neural tissue, and has revealed evidence of immune activation in the dorsolateral prefrontal cortex (PFC), orbital frontal cortex and midbrain of a substantial subset (~40–50%) of people with schizophrenia compared to a significantly smaller proportion (~0–10%) of age-matched non-schizophrenic controls [10, 11, 50–53]. These ‘high neuroinflammation’^1^ patients have been identified across independent cohorts using two-step recursive clustering of mRNA levels of several pro-inflammatory transcripts such as IL-1β, IL-6, IL-8 and SERPINA3 [50–53]. Given that structural and functional abnormalities in the dorsolateral PFC are hallmarks of schizophrenia [54–60], it is possible that inflammation in this region drives neuropathology—and potentially symptoms—in some patients. Indeed, the high neuroinflammation patient subgroup has worse psychotic symptoms [18], PFC-dependent cognition and neuropathology than patients with ‘normal’ levels of these transcripts, including reduced verbal fluency [19], increased astrogliosis [61], larger reductions in inhibitory interneuron-related transcripts [50, 62] and significant loss of prefrontal grey matter volume [51]. Recent evidence also suggests that this high neuroinflammation schizophrenia subgroup may have altered blood–brain barrier function that facilitates the trafficking of macrophages from blood to brain, evidenced by increased endothelial expression of adhesion molecules that capture white blood cells [63, 64]. Further, Cai et al. [63] and Purves-Tyson et al. [64] found elevated transcript levels of the macrophage marker CD163 mRNA in the PFC and midbrain of high neuroinflammation patients, supporting the contention that macrophages are recruited to the brain in response to cortical and subcortical immune activation. Taken together, these findings suggest that aberrant neuroinflammatory processes play a critical role in causing neuropathology in some patients.

The existence of inflammatory biotypes (subtypes) within schizophrenia may explain why studies measuring immune-related transcripts and/or proteins in the dorsolateral PFC of patients sometimes produce discrepant results. Many studies comparing dorsolateral PFC (and neighbouring PFC) tissue from people with schizophrenia as a single homogenous cohort to tissue from non-schizophrenic controls do find up-regulation of pro-inflammatory cytokines and acute phase proteins (IL-6, IL-8, TNFα, SERPINA3) and cytokine receptors (IL-1 receptor type 1 [IL1R1], TNF receptor 1 [TNFR1]), at the transcriptional level in schizophrenia [10, 50, 65–67]. IL-6 and TNFα have also been shown to be elevated at the protein level in this brain region in patients, along with another TNF family cytokine, lymphotoxin α [65]. Examination of cytokines that serve to dampen the inflammatory response has also revealed that the anti-inflammatory IL-10 transcript and protein are reduced in the dorsolateral PFC in schizophrenia [65], suggestive of a diminished ability to attenuate neuroinflammation in at least some patients. However, data from several postmortem studies dispute the role of neuroinflammation at both the molecular and cellular levels in schizophrenia [68–75]. Surprisingly, though, one such study reported significant enrichment of myeloid leucocyte activation in the DLPFC of people with schizophrenia relative to unaffected controls before concluding that immune activation is not a characteristic of the schizophrenia brain [68]. Notably, studies that fail to find positive associations between inflammatory markers in human postmortem brain tissue and schizophrenia did not investigate the possibility of heterogeneity in regards to immune subtypes. As such, immune ‘biotyping’ is an important research design tool that can be used to uncover previously unrealised neuropathology.

### Clinical evidence of inflammation in schizophrenia

Since immune-to-brain communication is bidirectional [76], assessing inflammation in the blood is useful in determining the extent of peripheral immune activation that may contribute to or result from neuroinflammation in schizophrenia. In fact, evidence for altered peripheral immune function predates evidence of increased brain cytokines [77–81]. More recently, elevations in inflammatory markers including pro-inflammatory cytokines, the acute phase protein CRP, the immune regulator S100B and soluble intracellular cell adhesion molecule have been found in the blood of people with schizophrenia [18, 19, 63, 82–97]. However, some studies have found unchanged [98–102] and even decreased [102, 103] levels of these same inflammatory biomarkers in the blood of patients compared to controls, which again may be due to elevations only occurring in a subset of patients. Similar to brain studies, studies of immune biomarkers in patient blood have produced conflicting results, though many strongly support peripheral inflammation in a subset of patients.

### Acute vs. chronic inflammation in schizophrenia

As consensus grows that inflammation plays a role in the pathophysiology of schizophrenia, important questions about the nature of this inflammation have arisen: Is inflammation consistent throughout a person with schizophrenia’s life or does it wax and wane? Further, is inflammation only apparent after the development of schizophrenia or can it be detected prior to the onset of symptoms? In terms of neuroinflammation specifically, it is difficult to assess fluctuations over the course of the illness. This is largely due to the inaccessibility of brain tissue and secretions to measure immune markers. As a result, neuroinflammatory status in living patients can only really be inferred from brain imaging studies or cerebrospinal fluid. Brain imaging studies often rely on radioligand-binding to the translocator protein (TSPO) to detect microgliosis and therefore neuroinflammation, and several studies have found increased TSPO binding in high-risk individuals, first episode psychosis and schizophrenia [104–106]. However, others have failed to find this effect in schizophrenia patients [107, 108]. TSPO is not specific for microglia [109–111], and there is inter-individual variability in TSPO binding in brain tissue for certain radioligands [112], complicating the interpretation and use of TSPO imaging. There have also been many reports of increased cytokines, immunoglobulin and antibody abnormalities and altered immune cell populations within the CSF of chronically ill people with schizophrenia as well as individuals with first episode psychosis [113–119]. These findings strongly suggest that neuroinflammation may be detected throughout the course of the illness. However, if schizophrenia was similar to other immune disorders, then we would expect inflammation to fluctuate over time. To the best of our knowledge, no study has directly compared CSF levels of immune markers between acutely and chronically ill patients over time.

Much insight into the course of (peripheral) inflammation in schizophrenia and its relationship to symptoms has been gained via assessment of immune factors in the blood of living patients. Inflammatory markers appear to fluctuate in tandem with symptom severity, peaking during times of acute psychosis and relapse. Acutely-ill patients have higher levels of both pro-inflammatory cytokines and CRP in their blood than those who are relatively stable [18, 120]. Evidence that inflammation actually precedes, rather than simply co-occurs with, the onset of psychosis comes from studies of high-risk individuals, where those who do go on to experience psychosis show elevations in blood inflammatory markers relative to low-risk individuals prior to transitioning [121, 122]. These findings also highlight the likelihood that immune disturbances in schizophrenia are not solely attributable to antipsychotic medications. In fact, several studies have found that some types of antipsychotic drugs can alleviate—but perhaps not fully normalise—inflammation in people with schizophrenia (reviewed in [123]). Despite this, even medicated patients who are chronically ill but clinically stable show evidence of inflammation in their blood [18, 19, 86, 90, 91, 97], albeit to a lesser degree than those experiencing acute psychosis. Overall, the evidence is convincing that inflammation is persistent yet also linked to symptom severity and clinical course in schizophrenia. Whether neuroinflammation in patients is chronic or transient (i.e. during acute psychosis), though, is less clear.

### Biotypes: identifying patients with inflammation-associated schizophrenia

Several researchers have attempted to stratify people with schizophrenia based on their immune status in the blood using either pro-inflammatory cytokine transcripts or CRP levels. When white blood cell cytokine mRNAs are used, ~40% of patients are deemed to have high peripheral inflammation [19, 90]. When CRP alone is used, the proportion of patients exhibiting peripheral inflammation varies from ~20–45% [16–18, 97, 124], and much of this variability may be attributable to the use of different cut-off values to define ‘high CRP’ as well as varying clinical status between patient cohorts (stable vs. acutely psychotic).^2^ Both methods of immune stratification have proven clinically informative: those defined as having high inflammation show more severe positive and negative symptoms, greater cognitive deficits, and more significant reductions in brain volume and cortical thickness [16–19, 97]. People with schizophrenia defined as being in the ‘high cytokine’ group have worse verbal learning and lower cortical grey matter volume in Broca’s area as compared to ‘low cytokine’ patients [19]. People who have both schizophrenia and high circulating CRP have more severe symptoms (psychiatric and cognitive) and reduced PFC thickness compared to low CRP patients [16–18, 97]. Therefore, peripheral inflammation in some people with schizophrenia appears linked to brain pathology (both structural and functional), and this relationship may be overlooked if the existence of inflammatory biotypes within schizophrenia is not considered.

In sum, examination of patient brain tissue and blood has revealed that inflammation is prevalent both within and outside of the brain in some people with schizophrenia, and that symptoms and morphological changes to the PFC may be more severe in this high inflammation subset of patients. Together, such findings support the contention that heightened inflammation exists in a significant number of people with schizophrenia and that the initiating pro-inflammatory signal could be body-derived or brain-derived. However, it has not yet been conclusively determined which markers of inflammation produce the most information about clinical course or severity, or which patient subgroups would be more likely to respond to which anti-inflammatory treatment. Thus, despite these recent advances in our molecular and cellular understanding of inflammation in the pathophysiology of schizophrenia, the expression of cytokines and acute phase proteins tells us little about the cause of inflammation in patients. Here, we consider that it may be more aetiologically informative to analyse inflammatory regulators upstream of cytokine and acute phase protein synthesis in patient subgroups, thereby identifying specific aspects of immunoregulation instead of inflammatory endpoints. In the next section, we will discuss evidence suggesting that NF-κB, one of the most significant immune regulators in the human body, is itself dysregulated in schizophrenia.

## Nuclear factor kappa B: a hub for immune regulation

Since many different physiological events can cause increased cytokines in brain (brain infection, neurodegenerative disease/tissue damage, ageing, neuronal stress), it is useful to determine if there are convergent or divergent changes in the molecular machinery controlling pro-inflammatory gene expression in the brain in schizophrenia. Further, there has long been consensus that schizophrenia has a strong genetic component and is a highly heterogenous disease, with only ~40% of patients showing evidence of neuroinflammation. Thus, we should consider potential pivotal points at which immune dysregulation and genetics may converge to ‘prime’ certain people for the development of inflammation-associated schizophrenia. Transcription factors are key molecules serving as genetic switches that change the phenotype and function of the cells in which they are expressed, including the control of cytokine gene expression. NF-κB is a family of five transcription factors that controls the expression of genes involved in the initiation, maintenance and termination of immune responses [125, 126] and is therefore considered a master regulator of inflammation. The overall level of NF-κB activity is regulated at the mRNA level [127–130], thus NF-κB is a transcription factor that is itself controlled by transcriptional cues within the cell.

Recently, dysregulation of the NF-κB pathway has been linked to schizophrenia [11, 12, 131], making NF-κB an attractive candidate for investigations into the cause of neuroimmune dysregulation in schizophrenia. The first study to examine NF-κB in the postmortem brains of people with schizophrenia without apparent neuroinflammation found that the entire NF-κB system was downregulated in patients in several brain regions, most notably in the temporal cortex [132]. However, more recently, overactivity of NF-κB in the dorsolateral PFC specifically has been linked to schizophrenia [11, 131] and we found that prefrontal cortical NF-κB dysregulation in a subset of patients appears to drive neuroinflammation in this region in these individuals [12]. These later findings align with overexpression of immune biomarkers that are under the control of NF-κB—such as IL-6, IL-1β, IL-8 TNFα and SERPINA3—in the brain and blood of patients [31–37, 50, 52, 53, 66, 133, 134]. Determining which specific aspects of NF-κB induction and/or inhibition are disrupted in people with inflammation-associated schizophrenia at the mRNA level may help us to better understand the cause of inflammation in these people. To appreciate the many ways in which NF-κB signalling may be disrupted by altered transcription of NF-κB-related mRNAs, it is necessary to first understand the structure and regulation of NF-κB.

### The structure of NF-κB dimers and their changed expression in the schizophrenia cortex

**Fig. 1:**
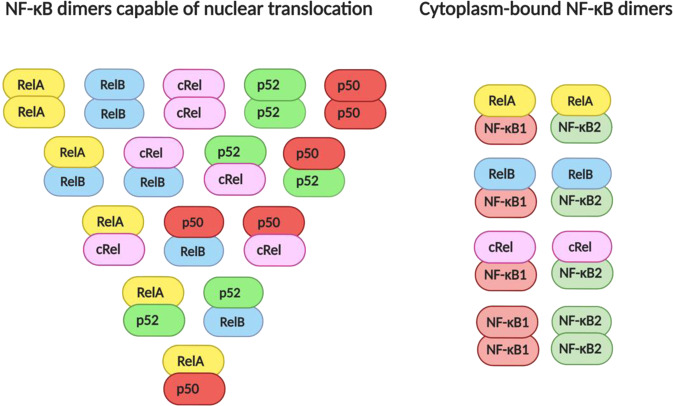
NF-κB dimers. NF-κB dimers may be made up of any two NF-κB subunits, however, unprocessed NF-κB1 and NF-κB2 retain Rel subunits in the cytoplasm.

NF-κB exists as homo- or hetero-dimers^3^ which are mostly sequestered in the cytoplasm until activated by pro-inflammatory stimuli. NF-κB dimers may be made up of any combination of five transcription factors (subunits) including RelA, RelB, cRel, NF-κB1 and NF-κB2, which readily dimerise in the cytoplasm. For an NF-κB dimer to be transcriptionally active, it must contain at least one of the Rel subunits, since these contain transactivation domains that allow the dimer to initiate transcription on upstream DNA regions of target genes [135]. In addition, NF-κB1 and NF-κB2 are synthesised as precursors and must be processed into mature subunits (p50 and p52, respectively) before translocating to the nucleus (Fig. 1). However, even p50 and p52 are transcriptionally inactive (and may even repress transcription of target genes) if bound to each other or themselves. Dimers containing RelA and cRel, most commonly bound to processed NF-κB1 (p50), are induced by the canonical pathway of NF-κB activation, while the dimer formed by RelB and processed NF-κB2 (p52) is induced by the non-canonical pathway of NF-κB activation. Though the DNA-binding affinities of dimers are largely overlapping [136], canonical NF-κB activation is rapid and typically transient, whereas activation of the non-canonical NF-κB pathway is characteristically slower and more persistent [137]. While the non-canonical NF-κB pathway is mainly involved in B-cell development and lymphoid organogenesis as opposed to acute immune responses orchestrated by the canonical pathway in immune cells [138], distinct functions of the two pathways are not well understood in the brain. However, the most abundant NF-κB dimers in mature glia contain RelA, suggesting the prime importance of NF-κB activation through the canonical pathway in microglia and astrocytes [139]. Though it has long been believed that neurons exhibit high basal levels of NF-κB reflective of a role for NF-κB in memory formation [140–145], more recent evidence points to very low NF-κB activity in cortical neurons both basally and after stimulation, but very high inducibility of NF-κB in cortical glia in inflammatory contexts [146, 147]. Thus, it appears that while neurons also possess the NF-κB ‘machinery’ to participate in neuroinflammation, they do so to a much lesser degree than glia.

**Fig. 2:**
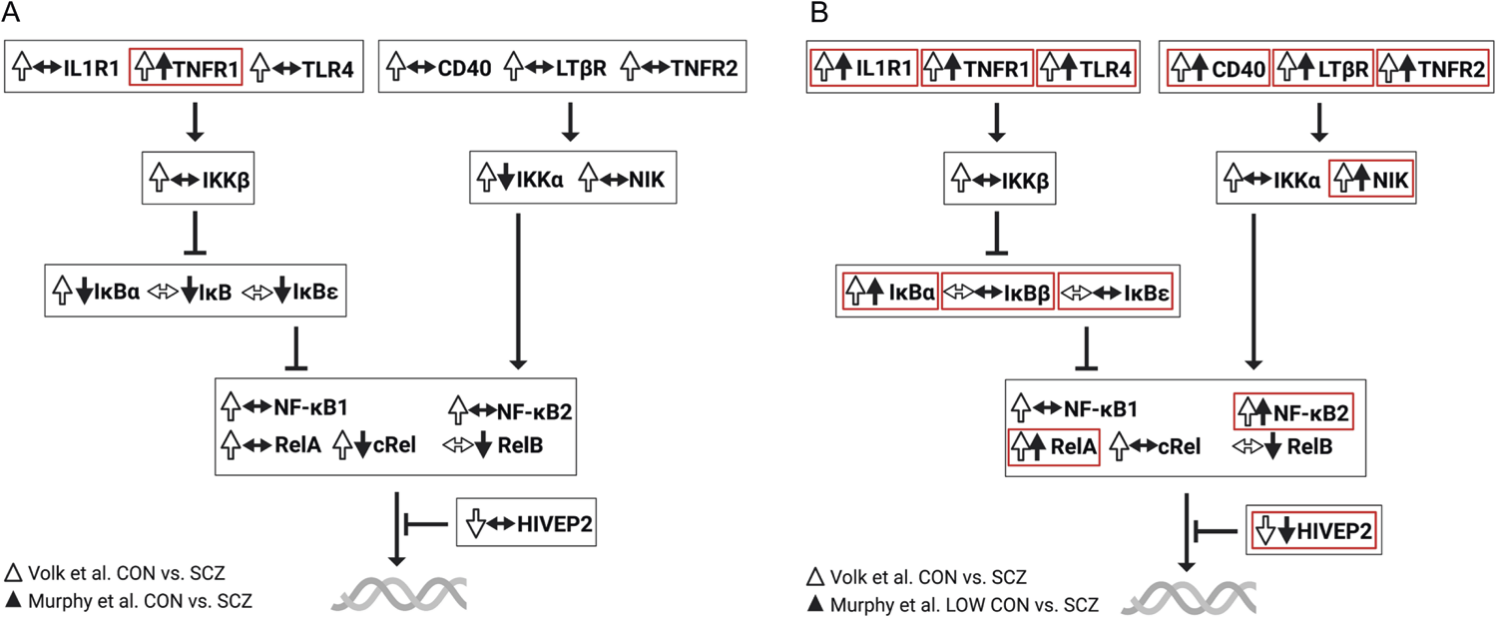
Expression of NF-κB transcripts in the DLPFC of controls vs. schizophrenia. Two studies have measured NF-κB pathway transcripts in the DLPFC of people with schizophrenia compared to unaffected, age-matched controls. **A** Volk et al. [11] report up-regulations at multiple levels of the pathway in schizophrenia while Murphy et al. [12] replicated only one of these findings (TNFR1) and found several down-regulations of the same NF-κB pathway transcripts in schizophrenia. **B** When high inflammation controls (individuals with elevated levels of pro-inflammatory mRNAs) were omitted from analyses in Murphy et al. [12], many (13 of 18) of the diagnostic comparisons in NF-κB pathway transcripts were consistent with those reported by Volk et al. [11]. Red boxes indicate shared findings between both studies.

NF-κB dimers binding to κB-binding sites on DNA promoters is the final step of NF-κB activation, and higher levels of subunit mRNA could logically lead to a higher rate of NF-κB dimer formation in the cytoplasm and hence a higher rate of pro-inflammatory gene expression. When considered as one homogenous group, people with schizophrenia have ~20–30% higher levels of RelA, cRel and NF-κB1 transcript and >80% higher levels of NF-κB2 transcript in the dorsolateral PFC compared to non-schizophrenic controls, while levels of RelB mRNA do not differ between the two groups [11]. However, we found that cRel was unchanged in people with schizophrenia, inflamed or not, despite being upregulated in high inflammation controls relative to low inflammation controls [12] (for comparison between the findings of Volk et al. and Murphy et al. refer to Fig. 2). Further, increases in RelA and NF-κB1 mRNAs may not occur in all patients but uniquely in the high inflammation patient subgroup [12]. In fact, it appears that RelA and NF-κB1 mRNAs are also upregulated in non-schizophrenic controls with increased inflammation and surprisingly, to an even greater degree than in high inflammation patients. By contrast, NF-κB2 mRNA is increased in people with inflammation to the same degree regardless of diagnosis (control or schizophrenia). This perhaps explains the greater magnitude of increase in NF-κB2 mRNA found when all people with schizophrenia are grouped together [11] as compared to RelA and NF-κB1 mRNA increases that would be less, on average, considering that non-inflamed people with schizophrenia make about 60% of the cases [50–52]. Taken together, these findings suggest that NF-κB activation (at least through the canonical pathway that activates the RelA/NF-κB1 heterodimer) may actually be blunted in people with schizophrenia compared to people without schizophrenia who have brain inflammation. Thus, this presents the intriguing possibility that there could actually be an inadequate neuroimmune response to physiological stress or tissue damage in people with schizophrenia. This is particularly interesting given the findings of Roussos et al. [132] that people with schizophrenia who do not appear to be in a state of neuroinflammation (the authors reported no change in several cytokine transcripts) show decreased expression of NF-κB pathway genes in the brain, particularly those molecules involved in the translocation of RelA. Lower basal NF-κB activity and/or a deficit in NF-κB activation may limit the capacity for inflammation to be resolved and explain the increases in pro-inflammatory cytokines observed by us and others. Therefore, it is possible that chronic low-grade inflammation in the dorsolateral PFC of people with schizophrenia continues unchecked due to inadequate NF-κB activation to meet a ‘threshold’ that triggers anti-inflammatory responses [148].

Contrary to the findings of Volk et al. we found that that RelB was downregulated in patients compared to controls overall [12]. RelB has been described as an ‘outlier’ in the NF-κB subunit family due to its several apparent anti-inflammatory roles (in addition to its dimer-forming, pro-inflammatory role) both in the nucleus and the cytoplasm [125], meaning less cortical RelB expression in people with schizophrenia could represent an additional failure to negatively regulate NF-κB. Overall, it appears that cortical inflammation in people with schizophrenia could be associated with a weaker-than-normal expression of the NF-κB subunits, such that levels may be sufficient to propagate, but not to ultimately shut off, the inflammatory response in brain.

Translocation of NF-κB dimers into the nucleus depends entirely on the status of upstream signals, since they cannot move from the cytoplasm without degradation of their bound inhibitors. Breakdown of NF-κB inhibitors is triggered by upstream signalling cascades. As such, without changes in NF-κB pathway regulators to facilitate dimer translocation, a higher number of NF-κB dimers in the cytoplasm may not necessarily mean higher NF-κB-mediated gene transcription. By working backwards from NF-κB in the pathway, we can examine which (if any) of the major NF-κB-activating cell-surface receptors and/or intracellular regulators may be changed in schizophrenia to add further support cytoplasmic release of NF-κB. This may provide clues as to which aspect(s) of the pathways are ultimately responsible for the drives NF-κB-related transcriptional increases in pro-inflammatory cytokines reported in schizophrenia.

### The central mechanism of NF-κB inhibition may be inadequate in the PFC in schizophrenia

In the absence of pro-inflammatory stimuli, canonical NF-κB dimers are bound to inhibitor of κB (IκB), masking their nuclear localisation sequence and retaining them in the cytoplasm [149, 150]. IκB protein can enter the nucleus and actively remove NF-κB dimers from DNA [151, 152]. Thus, IκB alpha (IκBα), IκB beta (IκBβ) and IκB epsilon (IκBε) are the major negative regulators of the canonical pathway. Further, genes encoding IκBs are targets of NF-κB itself and are upregulated by NF-κB activation and resynthesised post-stimulation to provide negative feedback [153, 154]. The transcription rate of IκBα in particular is considered a critical parameter that modulates NF-κB dynamics post-stimulation [155]. As such, elevated expression of IκBα is considered to be a proxy for increased NF-κB activity, and it has been reported that IκBα mRNA is increased in the dorsolateral PFC of people with schizophrenia relative to controls [11]. Unsurprisingly, though, IκBα is actually only upregulated in the subset of patients who also have elevated brain cytokines, similar to the situation for RelA and NF-κB1 mRNAs [12]. Further, stratifying cohorts based on neuroinflammatory status also revealed that IκBα mRNA levels are lower in high inflammation patients than in high inflammation controls [12], again pointing to lower-than-expected expression and/or induction of NF-κB regulatory transcripts. This putative deficit in NF-κB signalling could mean failure to attenuate inflammation in people with schizophrenia, thus compounding existing neuropathology.

### Further evidence of ‘blunted’ NF-κB activation in schizophrenia: altered expression of the regulatory transcript IKKβ

**Fig. 3:**
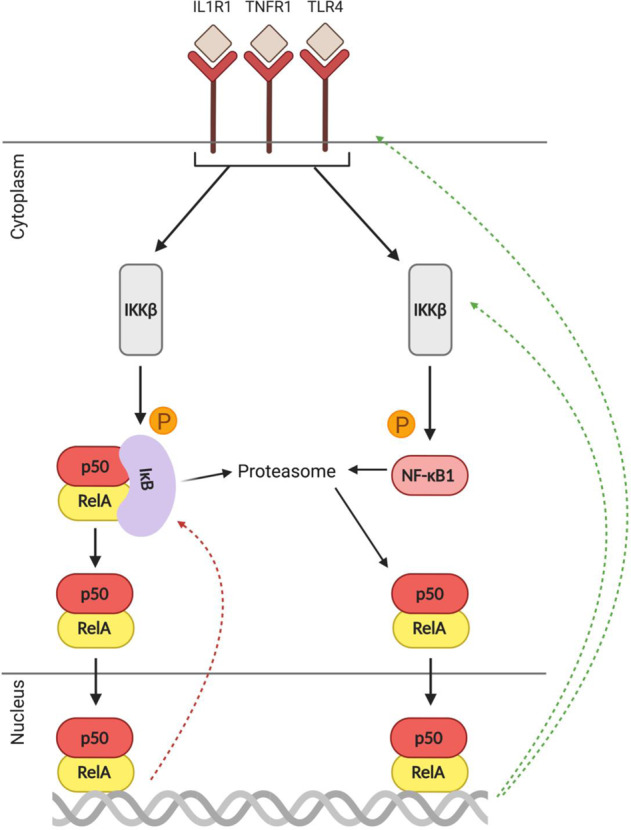
Regulation of canonical NF-κB activation. Cell-surface immunoreceptors activate IKKβ, which tags IκB for proteasomal degradation thereby freeing the p50-RelA/cRel dimer. Activation of canonical NF-κB receptors also enhances the partial processing of NF-κB1 into p50. As a result of NF-κB1 processing or IκBα degradation, the p50-RelA/cRel dimer moves into the nucleus where it initiates pro-inflammatory gene transcription. As such, IKKβ and IκB are considered the central regulators of the canonical pathway. Red dotted arrows indicate negative feedback via IκB transcription, green dotted arrows indicate positive feedback via receptor and IKKβ transcription.

Once canonical NF-κB-inducing receptors become activated at the cell surface, IκBs are targeted for degradation by the intracellular kinase inhibitor-of-NF-κB kinase subunit β (IKKβ). IKKβ phosphorylates IκBs leading to their degradation, and this process liberates NF-κB for nuclear translocation (Fig. 3). Increased IKKβ mRNA in the dorsolateral PFC has been reported in schizophrenia relative to controls [11], but may be more related to inflammation than to diagnosis and not unique to ‘inflamed’ patients but also occur in ‘inflamed’ controls [12]. However, similar to RelA and NF-κB1 mRNAs, levels of IKKβ transcript are significantly lower in high inflammation patients than in high inflammation controls [12]. Given that IKKβ is a chief positive regulator of canonical NF-κB, these findings provide further support for the theory that NF-κB activation may be blunted in people with schizophrenia compared to what a ‘normal’ response to increased brain cytokines would be.

### Expected levels of the non-canonical NF-κB regulatory transcript NIK in the PFC in schizophrenia

**Fig. 4:**
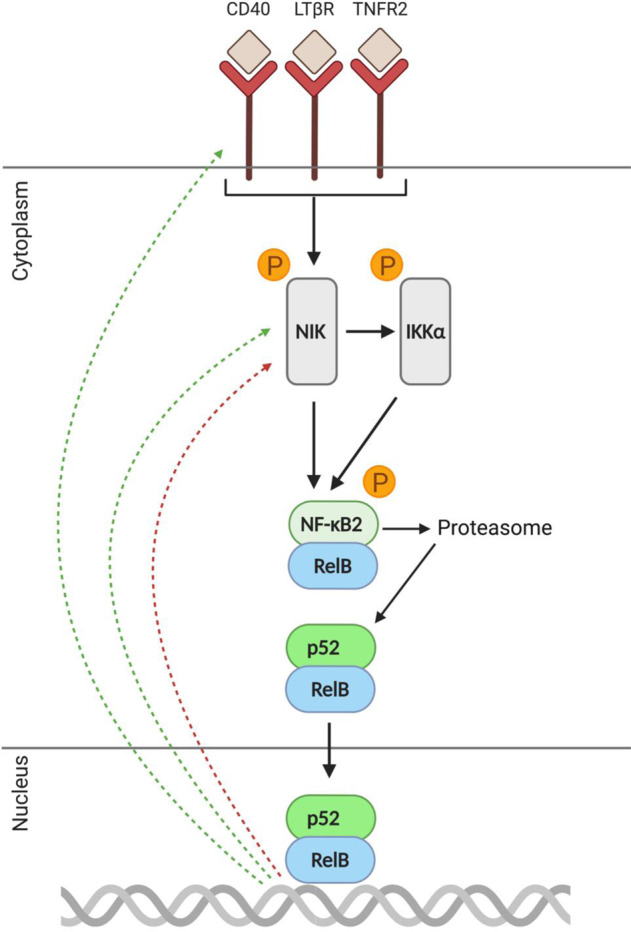
Regulators of non-canonical NF-κB activation. Cell-surface immunoreceptors activate NIK, which recruits IKKα to phosphorylate NF-κB2. NF-κB2 is then partially degraded by the proteasome into p52. As a result of NF-κB2 processing into p52, the p52-ReB dimer moves into the nucleus where it initiates pro-inflammatory gene transcription. As such, NIK is considered the central regulator of the non-canonical pathway. Red dotted arrow indicates negative feedback via NIK transcription, green dotted arrows indicate positive feedback via receptor and NIK transcription.

Similar to canonical dimers sequestered by IκB, the NF-κB2-RelB dimer of the non-canonical NF-κB pathway is also retained in the cytoplasm in the absence of pro-inflammatory stimuli (Fig. 4). Following activation of cell-surface non-canonical NF-κB-inducing receptors, NF-κB2 is partially processed by the proteasome into its mature subunit. This process is mediated by the actions of NF-κB-inducing kinase (NIK) and IKKα, which generate the active non-canonical p52-RelB dimer that then translocates to the nucleus and initiates target gene transcription. As such, NIK is the central regulatory kinase of the non-canonical pathway. NIK activity is mainly controlled post-translationally [156] but also appears to be regulated at the transcriptional level [157] and is upregulated in response to non-canonical NF-κB activation [158, 159]. Interestingly, unlike the regulatory kinase of the canonical pathway (IKKβ), NIK mRNA is elevated in high inflammation schizophrenia to the same degree as in those with inflammation who do not have schizophrenia [12]. That is to say, NIK up-regulation in the dorsolateral PFC appears to reflect a heightened neuroinflammatory state in general and is not specific to schizophrenia, contrary to previous interpretations of disease-specific increases when controls are viewed as one group (~90% of whom are likely non-inflamed [11, 50, 52].

### Focus on cell-surface receptors in schizophrenia: are changes in NF-κB-activating receptor mRNAs disease-specific?

Prior to kinases IKKβ and NIK liberating NF-κB dimers from their inhibitors, the first step of NF-κB induction is activation of cell-surface immunoreceptors. Several NF-κB -inducing receptor mRNAs are upregulated in the dorsolateral PFC in schizophrenia when examined by diagnosis [11], but stratification by inflammatory biotype indicates the majority of these changes are inflammation-specific and not disease-specific [12]. Therefore, changes in these cell-surface receptors tell us little about the specific pathways upstream of cytokine induction in schizophrenia, but instead suggest that it may be part of a more generalised brain reaction. The exception is the microglial-associated transcript TLR4, which is unchanged in high inflammation schizophrenia despite robust (approximately twofold) increases in high inflammation controls [12]. TLR4 mRNA is enriched in and highly functionally-relevant to microglia [160–163], as opposed to other canonical NF-κ-activating receptors such as IL1R1, the expression of which is unique to endothelial cells, astrocytes and some neurons but is not expressed by microglia at all under physiological conditions in mice [164]. Non-microglial cells have a limited TLR repertoire and TLR4 in particular appears largely unimportant for cells such as astrocytes, at least relative to its critical importance for the activation and effector functions of microglia [165]. The NF-κB subunits NF-κB1 and cRel also appear to be enriched in microglia [166] and as mentioned above, both of these transcripts were, like TLR4, lower in the cortex of high inflammation patients than high inflammation controls [12], further supporting a ‘blunting’ of canonical NF-κB activation in microglia in ‘inflamed’ patients. In light of conflicting reports regarding increased [11] and comparatively decreased [12] cortical TLR4 transcript levels in some patients, we posit that: (1) changes in TLR4 expression and microglial activation states in schizophrenia may be dynamic, and (2) neglecting to consider the neuroinflammatory status of individuals at their time of death when analysing immune-related transcripts in the brain may obscure the differences occurring in only a subset of patients.

Given that TLR4 is a potent activator of NF-κB and pro-inflammatory microglia [167], its putative suppression in the cortex in schizophrenia suggests that the increase in cytokines may not be microglia-derived and bolsters the theory of possible microglial suppression in a subset of patients. A putative lack of TLR4 expression by microglia also aligns with the above-mentioned blunting of transcriptional NF-κB1 in high inflammation patients since the abundance of NF-κB1 may coordinate the pro- to anti-inflammatory shift in microglia [168]. Together, these findings point to a lack of what may be physiologically appropriate pro-inflammatory microglia in patients with high levels of pro-inflammatory cytokines in the cortex.^4^

**Fig. 5:**
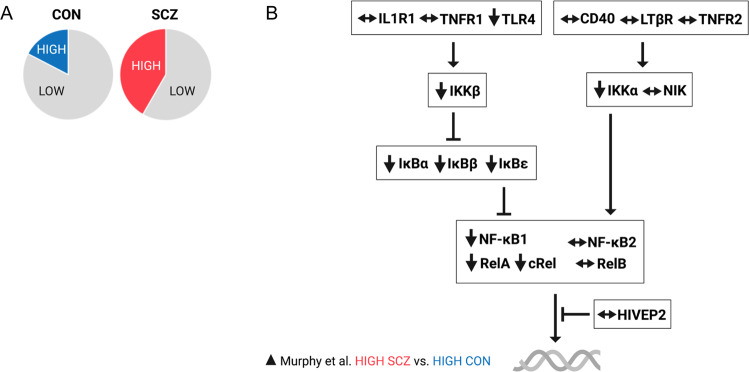
Expression of NF-κB transcripts in the postmortem DLPFC of schizophrenia patients with neuroinflammation relative to non-schizophrenic controls with neuroinflammation. **A** Not all patients show evidence of neuroinflammation (red = proportion of ‘inflamed’ patients, grey = non-inflamed patients), and some individuals without schizophrenia do show evidence of neuroinflammation (blue = ‘inflamed’ controls, grey = non-inflamed controls). Comparing cortical mRNA levels of NF-κB pathway members between high inflammation patients and high inflammation controls allows for identification of schizophrenia-specific abnormalities in this critical immunomodulatory pathway. **B** Such comparisons have shown that high neuroinflammation patients may actually have ‘blunted’ NF-κB activation, which may represent cell-specific deficits and/or a failure to adequately induce NF-κB to the level required to initiate NF-κB-dependent anti-inflammatory processes in the brain.

In contrast to TLR4, mRNA levels for one cytokine receptor that strongly induces the pro-inflammatory phenotype in astrocytes, IL1R1, are robustly increased in both high inflammation schizophrenia and high inflammation controls (see Fig. 5 for a summary of NF-κB pathway changes in high inflammation patients relative to high inflammation controls). Reactive astrocytes have been implicated in the inflamed patient subgroup previously [61], and are the major source of the inflammatory marker SERPINA3 that reliably identifies neuroinflammation across cohorts [50, 52, 66, 134, 170]. Together, these findings may indicate that normal microglial immune function via NF-κB is impaired in schizophrenia, and chronic NF-κB activation in non-microglial cells including astrocytes contribute to sustained, unimpeded cortical inflammation in some people with schizophrenia. It has even recently been proposed that reactive astrocytes release factors that keep microglia in a non-inflammatory state [171]; meaning inflammatory astrocytes and non-inflammatory microglia may mutually contribute to neuroinflammation in schizophrenia via their effects on each other. This is supported by studies finding evidence of reactive astrogliosis [53, 61, 131, 170, 172–175] and what appears to be paradoxical microglial suppression [63, 131, 176–179] in the PFC in at least some patients. However, it is important to note that some studies have failed to find evidence of astrogliosis [72–74, 180] while others have reported increased microgliosis in the PFC of people with schizophrenia [180, 181]. These conflicting results again highlight the neuropathological heterogeneity within the disease and the need for immune stratification to examine biologically distinct patient subgroups, but may also result from the use of different markers to identify glial pathology between studies. Future studies must consider the relative contributions of microglia, astrocytes and potentially even peripherally-derived immune cells to cortical inflammation in schizophrenia and to consider that these states are likely dynamic and not static.

## Conclusions, future directions and implications for treatment

The findings discussed above suggest that impairment in NF-κB regulation in the brain may be pathogenic in some people with schizophrenia. While most research has focused on endpoint markers of inflammation such as cytokines and acute phase proteins, identifying upstream mechanisms that regulate cytokine synthesis may provide greater insight and efficacy for novel therapeutics. The papers discussed in this review support the contention that a fault in microglial responses to immune stress in the dorsolateral PFC may contribute to prolonged pro-inflammatory responses from other cells such as astrocytes in patients [182]. General up-regulation of several NF-κB pathway transcripts in the PFC in patients with neuroinflammation may therefore reflect NF-κB activation in astrocytes and other non-microglial cells as opposed to microglia.

One important consideration here is that that weak NF-κB induction is not likely to represent a failure of the immune response in all contexts. In fact, the degree of NF-κB activation would logically be proportional to the magnitude of the inflammatory insult, thus less activation could just mean less stimulation. However, the putative NF-κB ‘blunting’ in high inflammation schizophrenia relative to high inflammation controls is curious and somewhat contradictory since high inflammation controls and high inflammation patients have the same degree of elevated inflammatory signalling in the cortex [50, 52], yet do not have the same levels of NF-κB transcripts that induce, and are induced by, these pro-inflammatory signals. This is why it is plausible that NF-κB is ‘normally’ activated in some cells such as astrocytes yet inappropriately underactive in other cells such as microglia in people with schizophrenia, while NF-κB is ‘normally’ activated in both cell types in high inflammation controls.

If overactivity of astrocytic NF-κB and underactivity of microglial NF-κB contribute to schizophrenia pathology, this presents a challenging hurdle for the development or repurposing of therapeutics to target NF-kB dysregulation in the brain. Blocking NF-κB in all cell types may worsen the problem by further suppressing microglia, but could also shut-off inflammatory signalling from astrocytes, thereby alleviating neuroinflammation and associated symptoms. This might be achieved by boosting levels of the chief NF-κB inhibitor, IκB, by blocking the action of IKKβ in the PFC. Indeed, several such compounds have been identified and show pre-clinical efficacy in the treatment of other inflammatory conditions including arthritis, chronic obstructive pulmonary disease and acute organ injury [183]. However, further dampening NF-κB activity in microglia may also have deleterious non-immune effects given its purported roles in brain homoeostasis and neuronal support [139, 184], which has also been reported in oligodendrocytes [185]. Thus, selective inhibition of NF-κB in astrocytes might be the most viable option for treating neuroinflammation in the brain in people with schizophrenia, and has proven neuroprotective in many mouse models of neuroinflammation. Inhibition of NF-κB in murine astrocytes post-CNS injury lessens pro-inflammatory cytokine production and neurodegeneration [186, 187], improves functional recovery [186], and substantially limits the extent of leucocyte infiltration to the damaged region [188]. Similarly in experimental autoimmune encephalitis, NF-κB signalling specifically in astrocytes contributes to a large degree of tissue damage and the influx of inflammatory immune cells into the CNS [189], a pertinent consideration given recent reports of increased numbers of leucocytes in the brains of some patients [63, 64, 190]. NF-κB activation in astrocytes has even been linked to memory impairment in a mouse model of dementia [191], underscoring the potential for astrocytic NF-κB to interfere with normal cognition. Peripherally, constitutive activity of NF-κB in myeloid cells has also been shown to drive pathogenicity of monocytes and macrophages during autoimmune-type neuroinflammation in mice [192]. Activation of NF-κB in macrophages specifically is thought to cause disruption of the blood–brain barrier, subsequent immune cell infiltration to the brain and resultant cognitive impairment and sickness behaviour which can be attenuated with peripheral NF-κB inhibition [193–195]. Given the several reports of increased pro-inflammatory, macrophage-derived cytokines in patient blood [19, 82–84, 90], increased TLR4 mRNA in patient white blood cells [196, 197] and exaggerated inflammatory responses to LPS (which activates NF-κB in macrophages via TLR4) in patient white blood cells [198], NF-κB in circulating monocytes may be an additional therapeutic target in schizophrenia. Several over-the-counter and prescription anti-inflammatory medications such as aspirin, minocycline and celecoxib are known to inhibit NF-κB in white blood cells (including macrophages) in vitro [199–201] and some early studies investigating the utility of these drugs in schizophrenia have produced promising results across various symptom domains [13, 202–204]. However, assessing the therapeutic benefit of anti-inflammatory drugs in schizophrenia based on patients’ immune status at commencement of treatment is yet to become a mainstream practice, which may lead to underestimation of treatment efficacy in ‘inflamed’ individuals, as mentioned above. Overall, future treatments targeting NF-κB in the PFC of people with schizophrenia must consider its cell-specific roles, and the possibility that targeting astrocytic NF-κB in the brain may be least likely to cause off-target, undesired effects on cells where NF-κB serves homoeostatic or even cell protective functions.

At present, the relative contributions of microglia, astrocytes, and even peripherally-derived immune cells to neuroinflammation in people with schizophrenia remains speculative since pro-inflammatory cytokine transcripts in the dorsolateral PFC have not been localised to specific cells (though general assessment of microgliosis and astrogliosis in postmortem patient brain tissue has been reviewed comprehensively elsewhere [205–208]). Further, the more detailed phenotypes of microglia in schizophrenia have not been determined and are likely dynamic, as exemplified by conflicting findings regarding TLR4 expression in the PFC in patients. Answering these remaining questions is crucial to the development of anti-inflammatory drugs to treat neuroinflammation in a subset of patients with schizophrenia, since it is clear that the normal healthy brain relies on NF-κB activation in the right cells, at the right time and to the right extent. Further thought and research will be required to develop an optimal therapeutic strategy to bring NF-κB activation signalling back into cellular and temporal homoeostasis in human brain.

## Footnotes

1 The terms ‘high neuroinflammation’ and ‘high inflammation’ used hereafter are relative to low/normal expression of immune markers and reflects subclinical inflammation in the absence of infection. These terms do not necessarily imply levels of inflammation comparable to acute infection or injury.

2 Evidence from first episode psychosis and chronically ill patients supports that peripheral inflammation fluctuates throughout the course of the illness and is highest during acute psychosis [18, 121].

3 Though NF-κB encompasses five separate transcription factors, the term ‘NF-κB’ is also commonly used to refer to the dimer complex comprised of two transcription factors. Thus, these transcription factors are also often referred to as NF-κB subunits. In this review, the term NF-κB will be used to describe the collective family of transcription factors, and separate transcription factors will be referred to as NF-κB subunits. The term ‘NF-κB activation’ will be used to describe the induction of pathways resulting in NF-κB dimer-DNA binding in the nucleus.

4 It is also important to note that some evidence supports an additional, non-immune role for microglial NF-κB in neuronal homoeostasis and hippocampal-dependent learning [169].
